# Supplementary material for: Comparative Analysis of Nucleic Acid-Binding Polymers as Potential Anti-Inflammatory Nanocarriers
Source: Pharmaceutics. 2023 Dec 20;16(1):10. doi: 10.3390/pharmaceutics16010010 (PMC10819575; doi:10.3390/pharmaceutics16010010)
Supplement: Supplementary file 1 [file pharmaceutics-16-00010-s001.zip › pharmaceutics-2711463-supplementary.pdf]

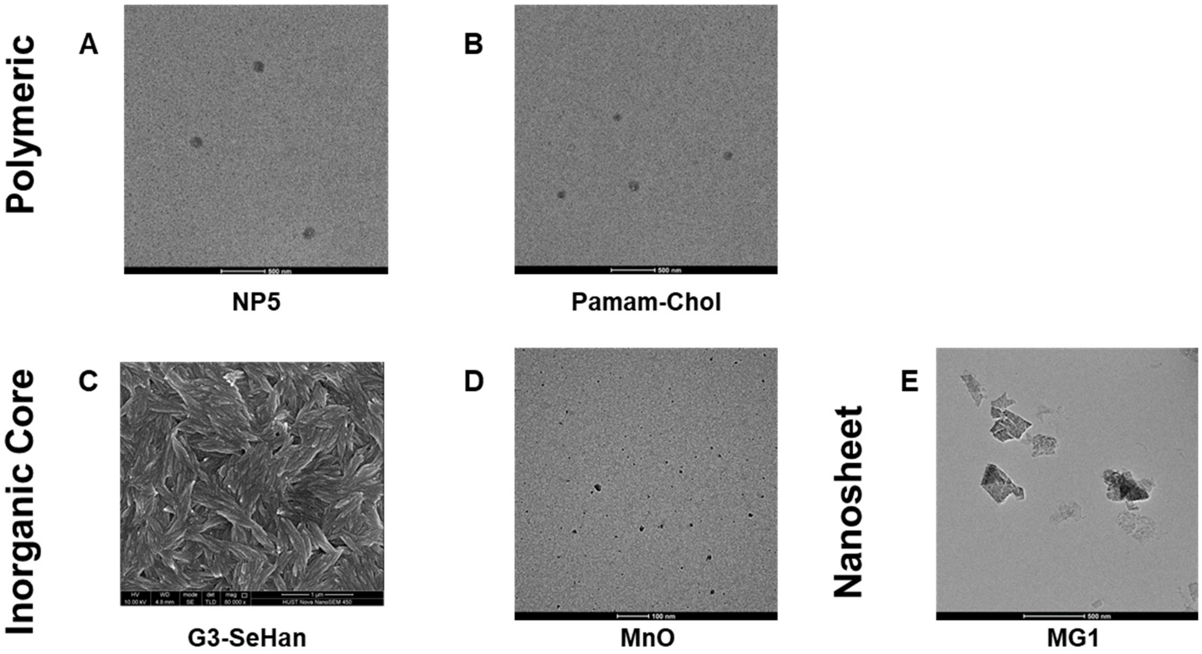

Figure S1. Electron Microscopy of NABNs. (A) SEM of Nanoparticle 5. (B) SEM of PAMAM-Chol Nanoparticles. (C) TEM of G3-SeHan Composite. (D) TEM of MnO particles. (E) TEM of MG1 2D Nanosheet.
